# Plant Growth-Promoting Rhizobacteria: Context, Mechanisms of Action, and Roadmap to Commercialization of Biostimulants for Sustainable Agriculture

**DOI:** 10.3389/fpls.2018.01473

**Published:** 2018-10-23

**Authors:** Rachel Backer, J. Stefan Rokem, Gayathri Ilangumaran, John Lamont, Dana Praslickova, Emily Ricci, Sowmyalakshmi Subramanian, Donald L. Smith

**Affiliations:** ^1^Department of Plant Science, McGill University, Montreal, QC, Canada; ^2^School of Medicine, Department of Microbiology and Molecular Genetics, Institute for Medical Research Israel-Canada, The Hebrew University of Jerusalem, Jerusalem, Israel

**Keywords:** phytomicrobiome, holobiont, rhizosphere, PGPR, sustainable agriculture, climate change resilience, roadmap, deployment

## Abstract

Microbes of the phytomicrobiome are associated with every plant tissue and, in combination with the plant form the holobiont. Plants regulate the composition and activity of their associated bacterial community carefully. These microbes provide a wide range of services and benefits to the plant; in return, the plant provides the microbial community with reduced carbon and other metabolites. Soils are generally a moist environment, rich in reduced carbon which supports extensive soil microbial communities. The rhizomicrobiome is of great importance to agriculture owing to the rich diversity of root exudates and plant cell debris that attract diverse and unique patterns of microbial colonization. Microbes of the rhizomicrobiome play key roles in nutrient acquisition and assimilation, improved soil texture, secreting, and modulating extracellular molecules such as hormones, secondary metabolites, antibiotics, and various signal compounds, all leading to enhancement of plant growth. The microbes and compounds they secrete constitute valuable biostimulants and play pivotal roles in modulating plant stress responses. Research has demonstrated that inoculating plants with plant-growth promoting rhizobacteria (PGPR) or treating plants with microbe-to-plant signal compounds can be an effective strategy to stimulate crop growth. Furthermore, these strategies can improve crop tolerance for the abiotic stresses (e.g., drought, heat, and salinity) likely to become more frequent as climate change conditions continue to develop. This discovery has resulted in multifunctional PGPR-based formulations for commercial agriculture, to minimize the use of synthetic fertilizers and agrochemicals. This review is an update about the role of PGPR in agriculture, from their collection to commercialization as low-cost commercial agricultural inputs. First, we introduce the concept and role of the phytomicrobiome and the agricultural context underlying food security in the 21st century. Next, mechanisms of plant growth promotion by PGPR are discussed, including signal exchange between plant roots and PGPR and how these relationships modulate plant abiotic stress responses via induced systemic resistance. On the application side, strategies are discussed to improve rhizosphere colonization by PGPR inoculants. The final sections of the paper describe the applications of PGPR in 21st century agriculture and the roadmap to commercialization of a PGPR-based technology.

## Introduction

A plant growing under field conditions is not an individual; it is a complex community (Lundberg et al., 2012) with subtle and relatively constant partner relationships. A well-structured and regulated community of microorganisms is always associated with the plant (Turner et al., 2013; Chaparro et al., 2014; Lebeis, 2014; Bulgarelli et al., 2015; Smith et al., 2015b). This community is the phytomicrobiome (Smith et al., 2017); the phytomicrobiome plus the plant is the holobiont (Berg et al., 2016; Theis et al., 2016; Smith et al., 2017). Microbiome relationships exist with all multi-cellular organisms, and probably all eukaryotes. In fact, these probably predate the colonization of the land by plants (Berg et al., 2014). This microbial community has been associated with terrestrial plants since their earliest evolution, to assist early land plants faced with challenges such as access to nutrients, novel and often-stressful conditions and pathogens (Smith et al., 2015a).

There are elements (including bacteria and fungi) of the phytomicrobiome associated will all major plant structures (flowers, fruits, stems, leaves, and roots) (Berg et al., 2016). However, conditions vary substantially among these structures, leading to specialized microbial populations inhabiting each one. The microbial community associated with the roots (the rhizomicrobiome), is the most populous and elaborate of all those associated with higher plants. The best understood and characterized example is the nitrogen-fixing rhizobia associated with legumes (Gray and Smith, 2005). Many members of the phytomicrobiome cannot be cultured and it has only been since the advent of metagenomics (Hirsch and Mauchline, 2012) and related methods that we are able to assess how membership is changed by conditions, plant genotype (Delaplace et al., 2015; Poli et al., 2016; Wintermans et al., 2016) and plant development.

The plant exerts considerable control over the composition of the rhizomicrobiome (Zhang et al., 2017). It produces root exudates of various compositions (Chaparro et al., 2012; Trabelsi and Mhamdi, 2013), which can be more suitable as a source of reduced C, to some microbes than others. The plant also produces signal compounds that recruit specific species and regulate their genetic and biochemical activities (Nelson and Sadowsky, 2015; Massalha et al., 2017; Smith et al., 2017). In addition, the soil microbial community undertakes various aspects of self-regulation (Leach et al., 2017). The microbes can produce quorum sensing compounds to communicate when conditions warrant a collective physiological shift (Chauhan et al., 2015). Plants have evolved to respond to microbial quorum sensing compounds and to produce analogs, providing plants with another level of regulation over the rhizomicrobiome (Ortiz-Castro et al., 2009). Finally, it is now becoming apparent that there is some degree of hierarchy within the phytomicrobiome and that there are key members, termed “hub species” (Agler et al., 2016) or “core species” (Toju et al., 2018), whose activities are regulated by plants, and hub species in turn regulate broader activities within the phytomicrobiome. Most hub species have probably been part of the phytomicrobiome for a very long time, allowing for development of their central position (van der Heijden and Hartmann, 2016).

**FIGURE 1:**
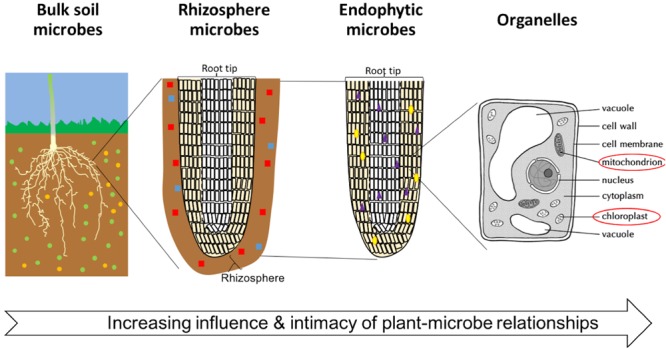
The degree of intimacy and influence of the plant-microbe interactions. Microbes are represented by small colored (red, green, yellow, purple, and blue) shapes. Diversity and number of microbes is variable between soils, distance from plant roots, crop species, and plant tissue.

In the soil, there is a gradient of intimacy between plant roots and microbes extending away from the plant root: the degree of plant influence over the microbial community increases nearer the root surface (Figure 1). This zone is now generally referred to as the rhizosphere, however, the term was originally coined by Hiltner (1904) to describe the soil microorganisms around and inside roots. Now, microbes living on the root surface are said to inhabit the rhizoplane, and those living inside the root are said to be endophytes (Gray and Smith, 2005; Zhang et al., 2017). Mitochondria and plastids (including the chloroplasts) represent some of the oldest and most intimate, aspects of the phytomicrobiome. They evolved from plant-associated microbes into the permanent subcellular structures we see today.

Our current understanding of the phytomicrobiome has demonstrated two main aspects. First, we know shockingly little about it (Quiza et al., 2015). Second, the relationships we have studied between rhizomicrobiome members and plants have shown that there is a tremendous potential in exploiting this community of organisms to increase worldwide crop production (Barea, 2015; Nehra and Choudhary, 2015; Smith et al., 2015b). This review is an update regarding the role of plant-growth promoting rhizobacteria (PGPR) in agriculture, from their collection to commercialization as a low-cost commercial agricultural input. While also we recognize the value of PGPR as a tool for phytoremediation, however, this is beyond the scope of our review; excellent information on this topic can be found in other review articles.

## Agricultural Context: a “Fresh” Green Revolution in the Face of Climate Change

The Green Revolution of the 20th century enabled unprecedented gains in global food production. The Green Revolution was roughly comprised of two main advances; chemical inputs (pesticides, herbicides, and chemical fertilizers) and improved crop plants (through targeted breeding and advanced genetic manipulations). However, gains associated with fertilizer inputs carry high environmental costs. A new revolution in agricultural innovation will be needed to sustain the food, fiber, and fuel needs of a growing global population and a changing climate through the 21st century. A “Fresh” Green Revolution, perhaps the Bio-Revolution, needs to be based on fewer intensive inputs with reduced environmental impact. A Bio-Revolution could be based on 1) biological inputs through utilization of the phytomicrobiome (with inoculants, microbially produced compounds, etc.), and improved crops (by manipulation of the phytomicrobiome community structure) (Timmusk et al., 2017). The use of microbial based agricultural inputs has a long history, beginning with broad-scale rhizobial inoculation of legumes in the early 20th century (Desbrosses and Stougaard, 2011). More recently, strains of *Bacillus, Pseudomonas, Glomus*, and others have been commercialized. The use of bacterial taxa in plant production has been reviewed previously for *Bacillus* (Borriss, 2011), *Pseudomonas* (Santoyo et al., 2012; Sivasakthi et al., 2014), *Actinobacteria* (Shivlata and Satyanarayana, 2017), and *Lactobacillus* (Lamont et al., 2017). In addition, *Acetobacter, Azospirillum, Paenibacillus, Serratia, Burkholderia, Herbaspirillum*, and *Rhodococcus* have also been shown to enhance crop production (Babalola, 2010).

The effects of climate change are expected to impose more environmental stresses on crops worldwide (Pachauri et al., 2014). Moreover, as climate change progresses throughout the 21st century, significant areas of high-quality agricultural lands will likely be lost to rising seas, erosion, salinization, and desertification. This means that crop yields will need to be maintained, in spite of production on a smaller area of land, under more stressful conditions. The phytomicrobiome plays a critical role in the survival of the holobiont, particularly for plants growing in extreme environments. Some plants that live in hypersaline coastal environments or geothermal soils rely on endophytic fungi to survive (Rodriguez and Redman, 2008). Likewise, constitutive microbial communities of agave (Coleman-Derr et al., 2016) and cacti (Fonseca-García et al., 2016) likely aid in the survival of these plants in very dry habitats. The microbiomes of plants native to extreme environments may be rich sources of stress-ameliorating microbes.

## Physiology of Plant-Growth Promoting Rhizobacteria

Plant-microbe co-evolution has led to some of the bacteria becoming facultative intracellular endophytes (Bulgarelli et al., 2013). Among these free-living bacteria are PGPR that exert beneficial effects on plants through direct and indirect mechanisms. Beneficial rhizobacteria have been utilized to improve water and nutrient uptake, abiotic and biotic stress tolerance. Even though numerous soil bacteria have been reported to promote plant growth and development, the mode(s) of action by which the bacteria exhibit beneficial activities are often not well understood. The molecular basis of plant-bacteria interaction mechanisms responsible for the physiological changes are beginning to be discerned, mainly due to the emerging “omics” approaches.

### Nutrient Acquisition by PGPR

Soils with dynamic microbial ecologies and high organic matter typically have lower fertilizer requirements than conventionally managed soils (Bender et al., 2016). For example, bulk microbial activity in soils is often considered when managing the application of organic nutrient sources. Phytomicrobiome research is beginning to reveal specific plant-microbe interactions that directly aid in plant nutrition (Beattie, 2015). Microbes that assist in plant nutrient acquisition (biofertilizers) act through a variety of mechanisms including augmenting surface area accessed by plant roots, nitrogen fixation, P-solubilization, siderophore production and HCN production (Pii et al., 2015). Therefore, manipulating microbial activity has great potential to provide crops with nutritional requirements.

The most extensively studied and exploited beneficial plant-bacteria relationship is the N-fixing symbiosis between rhizobia and legumes. In this relationship, legumes provide rhizobia with reduced C and a protected, anaerobic environment required for nitrogenase activity, while rhizobia provide the legumes with biologically available N. Within this symbiosis, both rhizobia and legume undergo significant transformations. The legume forms a new organ, the nodule, to house the rhizobia, and the rhizobia, in turn, changes from its free-living rod-shaped cell type to a branched, N-fixing bacteroid (Oke and Long, 1999). Rhizobial N-fixation contributes significant amounts of N to global agricultural systems, with estimates ranging from 20 to 22 Tg N per year (Herridge et al., 2008) up to 40 Tg N per year (Galloway et al., 2008). Rhizobial inoculants of leguminous crops are the earliest example of commercial microbial products in agriculture and still represent the most widely used agricultural inoculants (Bashan, 1998). However, genetic improvements in efficiency of the N-fixing symbiosis of rhizobia and crop plants have been elusive. The fixation of atmospheric nitrogen and conversion to ammonia is an energy demanding process, which means oxidative phosphorylation of carbon sources to generate ATP must be favored over glycogen synthesis within the bacterial cell, to increase nitrogen fixation. However, experiments with glycogen synthase deletion mutants of *Rhizobium tropici* have not survived in soil environments, despite increased dry matter and nodule number in inoculated bean plants (Marroquí et al., 2001).

Starting in the early 21st century, interest began to mount around the development of commercial inoculants of free-living N-fixing bacteria such as *Azoarcus* sp., *Burkholderia* sp., *Gluconacetobacter* sp., *Diazotrophicus* sp., *Herbaspirillum* sp., *Azotobacter* sp., *Bacillus polymyxa*, and especially *Azospirillum* sp. (Vessey, 2003). These free-living diazotrophs provide N to a much wider range of crop plants than rhizobia. Commercial inoculants of *Azospirillum*, produced by small and medium sized companies around the world, have been effective in increasing the yield of various cereal crops (Bashan and de-Bashan, 2015). Other bacteria that do not fix N have been shown to increase N uptake in plants, thus increasing nitrogen use efficiency (Adesemoye et al., 2008; Adesemoye and Kloepper, 2009), likely due to increased root growth, which allows plants to access more soil (Beattie, 2015).

Following Liebig’s law of the minimum in mind, the next most limiting nutrient for crop plants after N is usually P. While most agricultural soils contain ample quantities of P, much of it is in non-soluble forms. To supplement indigenous soil P, crops are typically fertilized with rock phosphate mined from one of a few large deposits (up to 85% of the world’s rock phosphate is estimated to be in Morocco and Western Sahara). Furthermore, phosphorus solubilizing microorganisms (PSMs) can help plants access the reservoir of non-labile phosphorus by releasing it from its recalcitrant forms. Inorganic P complexed with Ca, Fe, or Al can be solubilized by organic acids or H^+^ ions excreted by PSMs. Similarly, phytase produced by PSMs can liberate reactive P from organic compounds. Production of HCN by PGPR was originally thought to promote plant growth by suppressing pathogens, however, this idea has recently been challenged by Rijavec and Lapanje (2016), who argued that HCN indirectly increases P availability by metal chelation and sequestration of these geochemical entities. PSMs produce organic acids to reduce metal toxicity by using these compounds to transform metal species to immobile forms or chelate them for mobility, to be carried into the plant tissues for further phyto-extraction possibilities (Ahemad, 2015). The PSM *Bacillus megaterium* has been commercialized as BioPhos (BioPower Lanka, Sri Lanka) and can reduce phosphate fertilizer requirements of plantation crops up to 75% (Mehnaz, 2016). Strains of P- solublizing *Pseudomonas striata, B. Polymyxa*, and *B. megaterium* have also been commercialized by AgriLife (India) (Mehnaz, 2016).

Other nutrient elements, such as Fe and Zn can limit crop yields. Like P, Fe can also be abundant in soils, but unavailable to plants. Many bacterial strains increase the availability of Fe through the production of organic acids or siderophores (Kloepper et al., 1980; Neilands, 1995; Ahmed and Holmstrom, 2014). Siderophores also act to control pathogenic microbes by depriving them Fe (Ahmed and Holmstrom, 2014; Saha et al., 2016). A commercial formulation of the Fe mobilizing bacteria, *Acidithiobacillus ferrooxidans* has been developed by AgriLife (India) (Mehnaz, 2016), although this genus apparently solubilizes Fe through organic acid production rather than with siderophores (Bhatti and Yawar, 2010). Several strains of Zn-mobilizing bacteria have been shown to increase Zn uptake, and thus increase yield in several crops, including rice (Tariq et al., 2007; Shakeel et al., 2015), wheat and soybean (Ramesh et al., 2014). While the mechanisms of Zn-mobilizers remain uncertain, they are likely similar to those of PSMs and Fe-mobilizers, namely the production of chelating agents and organic acids (Hafeez et al., 2013).

### Signal Exchange Between Plant Roots and PGPR

#### Plant Hormones Produced by PGPR

Phytohormones are key players in regulating plant growth and development. They also function as molecular signals in response to environmental factors that otherwise limit plant growth or become lethal when uncontrolled (Fahad et al., 2015). Many rhizosphere bacteria are known to excrete hormones for root uptake or manipulate hormone balance in the plants to boost growth and stress response.

Many PGPR can produce auxins (Omer et al., 2004; Gupta et al., 2015) to exert particularly strong effects on root growth (Jha and Saraf, 2015) and architecture (Vacheron et al., 2013). Indole-3-acetic acid (IAA) is the most widely studied auxin produced by PGPR. It is involved in plant-microbe interactions (e.g., Ahemad and Kibret, 2014; Afzal et al., 2015). The function of exogenous IAA is dependent on the endogenous IAA levels in plants. At optimal IAA concentrations in plants, application of bacterial IAA may have neutral, positive, or negative effects on plant growth (Spaepen and Vanderleyden, 2011). PGPR that produce auxins have been shown to elicit transcriptional changes in hormone, defense-related, and cell wall related genes (Spaepen et al., 2014), induce longer roots (Hong et al., 1991), increase root biomass and decrease stomata size and density (Llorente et al., 2016), and activate auxin response genes that enhance plant growth (Ruzzi and Aroca, 2015).

Many PGPR produce cytokinins and gibberellins (Gupta et al., 2015; Kumar et al., 2015) but the role of bacterially synthesized hormones in plants, and bacterial mechanism of synthesis, are not yet completely understood (Garcia de Salamone et al., 2001; Kang et al., 2009). Some strains of PGPR can promote relatively large amounts of gibberellins, leading to enhanced plant shoot growth (Jha and Saraf, 2015). Interactions of these hormones with auxins can alter root architecture (Vacheron et al., 2013). Production of cytokinins by PGPR can also lead to enhanced root exudate production by the plant (Ruzzi and Aroca, 2015) potentially increasing the presence of PGPR associated with the plant.

Ethylene is a gaseous hormone, active at extremely low concentrations (0.05 mL L^-1^) and is a “stress hormone,” as illustrated by its concentration spiking during various abiotic and biotic stresses. Accumulation of ethylene in response to stress may increase plant tolerance or exacerbate stress-response symptoms and senescence (Morgan and Drew, 1997). PGPR function has been studied under both stressed and unstressed conditions and often provides greater growth stimulation under stressful conditions, for instance, under drought stress (Rubin et al., 2017). Ethylene plays an important role for improving plant stress tolerance for some PGPR (Nadeem et al., 2014): PGPR secrete 1-aminocyclopropane-1-carboxylase (ACC) deaminase which reduces ethylene production in plants (Glick, 2014; Vejan et al., 2016). Many studies have shown enhanced stress tolerance in plants through inoculation with PGPR that produce ACC deaminase. This appears to occur since PGPR are able to keep ethylene levels from reaching levels sufficient to reduce plant growth (Ahemad and Kibret, 2014; Pérez-Montaño et al., 2014; Ruzzi and Aroca, 2015), as has been demonstrated with *Camelina sativa* (Heydarian et al., 2016).

#### Other Microbe-to-Plant Signal Molecules

A wide range of secondary metabolites and volatile organic compounds (VOCs) produced by bacteria can improve stress-tolerance and/or stimulate growth in plants. For example, polyamines play important physiological and protective roles in plants. *B. megaterium* BOFC15 secretes a polyamine, spermidine, and induces polyamine production in *Arabidopsis*, resulting in an increase in biomass, altered root architecture and elevated photosynthetic capacity. The inoculated plants exhibited higher drought tolerance and abscisic acid (ABA) content under PEG induced water-deficit stress (Zhou et al., 2016). A range of PGPR produce HCN, which can control the level of deleterious microbes in the rhizosphere (Kumar et al., 2015). VOC produced by PGPR stimulate plant growth, resulting in increased shoot biomass and improve plant stress resistance (Bailly and Weisskopf, 2012; Ruzzi and Aroca, 2015).

The microbes of the phytomicrobiome also affect each other’s activities through signal compounds (Hagai et al., 2014; Massalha et al., 2017). These signals amount to hormones of the holobiont. For example, lumichrome and riboflavin can act as microbe-to-plant signal compounds able to stimulate plant growth. Both compounds can cause meaningful alterations in plant development; lumichrome can accelerate appearance of leaves (more rapid development) and leaf expansion (enhanced growth). In addition, it can increase plant height and overall leaf area, resulting in improved production of biomass. This is true over a wide range of plant types including both monocots and dicots (Dakora et al., 2015).

Microbe-to-plant signal compounds (e.g., lipo-chitooligosaccharides and thuricin 17) have been shown to increase plant growth for diverse species, particularly when plants are growing under stressful conditions (Subramanian and Smith, 2015; Subramanian et al., 2016b; Zipfel and Oldroyd, 2017). The receptor for the lipo-chitooligosaccharides is a LysM kinase for the legume-rhizobia symbioses; this receptor system seems to have evolved for pathogen detection almost Two billion years ago (Spaink, 2004; Gust et al., 2012; Carotenuto et al., 2017). The microbe-to-plant signal in the N_2_-fixing *Frankia* symbiosis remains to be identified but appears not to be an LCO (Chabaud et al., 2016).

#### Root Exudates as Plant-to-Microbe Signals

Plants excrete considerable control over the microbes they associated with (Berendsen et al., 2012; Badri et al., 2013; Turner et al., 2013; Massalha et al., 2017); even of simple genotype differences within a plant species can have meaningful effects (Peiffer et al., 2013; Winston et al., 2014). Some of this control is the result of inter-organismal signals (Smith et al., 2017). Starting when the seed is imbibing and germinating, then when roots are growing and finally senescing, molecules are released from roots into the surrounding soil. These molecules support microbial growth and activity in the rhizosphere (Nelson, 2004a, 2017; Schiltz et al., 2015). Variation in root exudation (timing, amount, and/or constituents) provides a mechanism by which plants can manipulate composition and abundances of their root-associated microbiota (Bakker et al., 2012). Exudates are thought to consist mainly of sugars, amino acids, and organic acids that are present at high concentrations in the cytoplasm of the plant, but also include smaller amounts of complex secondary metabolites such as flavonoids, terpenes, and phenolic compounds that can attract specific microbes in the rhizosphere (Jones et al., 2004; Bais et al., 2006; Musilova et al., 2016). It has also been suggested that exudation of the signal molecules jasmonic acid and salicylic acid into the rhizosphere can be involved in the interplay between roots and microbes during the initial events of colonization (Gutjahr and Paszkowski, 2009; Doornbos et al., 2011). Root exudation is genetically regulated and can thus shape distinct rhizobacterial communities for different plant genotypes, resulting in highly variable exudates among plant species, individual plant types within the same species, at different plant developmental stages, growth conditions, and biotic interactions (Gransee and Wittenmayer, 2000; Mougel et al., 2006; Broeckling et al., 2008; Houlden et al., 2008; Badri and Vivanco, 2009; Micallef et al., 2009; Badri et al., 2013; Kristin and Miranda, 2013).

## PGPR Improve Plant Growth Under Stressful Growing Conditions

The mechanisms that regulate stress tolerance in plants are intricate and complex, in part because plants are sessile organisms (Wani et al., 2016) which have no choice but to stand where they are and “take it.” Improving stress tolerance in crop plants through conventional breeding is a long and capital-intensive process, while genetic engineering is associated with ethical and social acceptance issues. The role of beneficial microorganisms is gaining importance in stress management and the development of climate change resilient agriculture. Recent studies have exploited molecular techniques to understand the mode of action of the plant-microbe interactions resulting in induced stress tolerance.

### Abiotic Stress Tolerance Associated With PGPR

*Pseudomonas putida* MTCC5279 ameliorated drought stress in chickpea (*Cicer arietinum*) plants by modulating membrane integrity, osmolyte accumulation (proline, glycine betaine) and ROS scavenging ability. Stress responses were positively modulated by the bacteria resulting in differential expression of genes involved in ethylene biosynthesis (ACO and ACS), salicylic acid (PR1), jasmonate (MYC2) transcription activation, SOD, CAT, APX, and GST (code for antioxidant enzymes), DREB1A (dehydration responsive element binding), NAC1 (transcription factors expressed under abiotic stress), LEA and DHN (dehydrins) (Tiwari et al., 2016). Application of thuricin 17 produced by *Bacillus thuringiensis* NEB17 to soybean (*Glycine max*) under water-deficit conditions resulted in modification of root structures and increased root and nodule biomass, root length, root ABA, and total nitrogen content (Prudent et al., 2015). Beneficial microbes also help plants cope with flooding stress. Rice (*Oryza sativa*) seedlings inoculated with an ACC deaminase producing strain of *Pseudomonas fluorescens* REN1 increased root elongation under constantly flooded conditions (Etesami et al., 2014).

Salt stress effects can be diminished by ACC deaminase. Pea plants inoculated with *Variovorax paradoxus* 5C-2, which produce ACC deaminase, had increased photosynthetic rate, electron transport, balanced ion homeostasis through increased K^+^ flow to shoots and Na^+^ deposition on roots, decreased stomatal resistance and xylem balance pressure and increased biomass under salt stress at 70 and 130 mM NaCl (Wang et al., 2016). For okra, PGPR producing ACC enhanced salt tolerance, increased antioxidant enzyme activities (SOD, APX, and CAT) and upregulated ROS pathway genes (CAT, APX, GR, and DHAR) (Habib et al., 2016). Maize seedlings inoculated with *Bacillus amyloliquefaciens* SQR9, had enhanced salt stress tolerance, including enhanced the chlorophyll content, compared with the control. Additional analysis showed that the mechanisms were related to enhanced total soluble sugar content leading to decreased cell destruction, improved peroxidase/catalase activity and glutathione content for scavenging ROS, and reduced Na^+^ levels in the plant. These physiological manifestations were confirmed by measured upregulation of RBCS, RBCL, H^+^ -PPase, HKT1, NHX1, NHX2, and NHX3 genes, as well as downregulation of NCED expression, as determined by qPCR (Chen et al., 2016). Wheat (*Triticum aestivum*) plants inoculated with the halotolerant *Dietzia natronolimnaea* showed upregulation of genes involved in the ABA-signaling cascade, salt overly sensitive (SOS) pathway, ion transporters, and antioxidant enzymes; stress tolerance is induced by modulation of complex network of gene families (Bharti et al., 2016).

Exposure to cold and/or heat reduce yield and, in worst case scenarios, result in crop failure (Cheng, 2014). A gibberellin-producing PGPR, *Serratia nematodiphila* increases pepper (*Capsicum annum*) growth under low temperature stress conditions. The inoculated plants contained more GA4 and ABA and less salicylate and jasmonate (Kang et al., 2015). Inoculation with *Burkholderia phytofirmans* PsJN modulated carbohydrate metabolism to reduce chilling damage to grapevine (*Vitis vinifera*) plantlets exposed to low temperature stress (Fernandez et al., 2012). Inoculation of tomato (*Solanum lycopersicum*) plants exposed to low temperatures with *Pseudomonas vancouverensis* OB155 and *P. frederiksbergensis* OS261 increased expression of cold acclimation genes and antioxidant activity in leaf tissues (Subramanian et al., 2015).

### Biocontrol and Induced Systemic Resistance for Biotic Stress Tolerance

*Bacillus amyloliquefaciens* (SN13) is a biocontrol agent against *Rhizoctonia solani*, by prolonging tolerance through enhanced defense response in the plants. The colonized plants exhibit modulation of phytohormone signaling, sustained maintenance of elicitors, production of secondary metabolites and balance of reactive oxygen species and scavengers producing ROS scavengers (Srivastava et al., 2016). Cotton (*Gossypium hirsutum*) plants inoculated with *Bacillus* spp. exhibited increased gossypol and jasmonic acid secretion reducing larval feeding by *Spodoptera exigua*. Transcript levels of genes involved in synthesis of allelochemicals and jasmonates were higher in inoculated plants as was suppression of the pest (Zebelo et al., 2016). *Enterobacter asburiae* BQ9 induced resistance against tomato yellow leaf curl virus by increasing the expression of defense-related genes and antioxidant enzymes, including phenylalanine ammonia lyase, peroxidase, catalase, and superoxide dismutase (Li et al., 2016). Soil inoculation with *Peanibacillus lentimorbus* B-30488 decreased cucumber mosaic virus RNA accumulation in *Nicotiana tabacum* cv. White burley leaves by 91%. This was associated with an increase in stress and pathogenesis-related gene expression and antioxidant enzyme activity suggesting induced resistance against the virus. PGPR colonization resulted in improved tissue heath and physiology of plants, which produced more flowers and seeds (Kumar et al., 2016). The bacteria also produce ACC deaminase and induce tolerance against southern blight disease in tomato caused by *Scelerotium rolfsii*. The inoculated plants showed modulation of the ethylene pathway and antioxidant enzyme activities; systemic tolerance was corroborated by pathogen related gene expression analysis (Dixit et al., 2016). Acyl-homoserine lactones (AHL)-producing *Serratia liquefaciens* MG1 and *P. putida* IsoF elicited induced systemic resistance (ISR) in tomato (*S. lycopersicum*) against *Alternaria alternate* whereas AHL-null mutant strains of both PGPR resulted in reduced ISR (Schuhegger et al., 2006). Root exudates have been found to contain chemicals that mimic AHL signals, stimulating beneficial rhizosphere associations while inhibiting pathogenic bacteria (Teplitski et al., 2000).

Besides functioning as biocontrol agents, PGPR protect plants against pathogens by eliciting biochemical and molecular defense responses within the plant (Lugtenberg and Kamilova, 2009). PGPR can trigger ISR in plants, which activates pathogenesis-related genes, mediated by phytohormone signaling pathways and defense regulatory proteins to prime plants against future pathogen attack (Pieterse et al., 2014). Bacterial signal compounds and microbe-associated molecular triggers, such as chitin oligomers, have been shown to modulate ISR induction in plants. Pathogen cell-surface factors such as flagellins and *O*-antigen of lipopolysaccharides elicit ISR, whereas analogs of salicylic acid and jasmonic acid trigger ethylene to elicit NPR1 mediated systemic acquired resistance (SAR) in plants (Ping and Boland, 2004).

## Strategies for Improving Rhizosphere Colonization by PGPR Inoculants

Under field conditions, other external factors come into play and the ability of soil bacteria to elicit positive effects on plant growth can be impaired and so that the effects of applying specific PGPM can be variable (Nelson, 2004b). The plant rhizosphere is colonized by microorganisms from the soil and the seed. The determinants of soil microorganisms are based on properties such as C and N availability, organic matter content, water availability and pH (Bossio et al., 1998; Drenovsky et al., 2004; Garcia-Pausas and Paterson, 2011) as well as biogeographic patterns including soil type and seasonality (Kristin and Miranda, 2013). Hence it is necessary to develop strategies for effective inoculation methods, so that bacteria of interest gain advantage in colonization efficiency over others. Product quality, compatibility, and stability determine effective colonization and consistent performance of the inoculum under field conditions (Lee et al., 2016).

### Biofilm Versus Planktonic Inoculum

Plant-associated biofilms have been shown to establish themselves on various parts of plants such as leaves, roots, seeds and internal vasculature (Ramey et al., 2004; Ude et al., 2006; Danhorn and Fuqua, 2007; Eberl et al., 2007). The ability to form biofilms not only enhances bacterial survival but also enhances plant growth through the various PGPR-associated mechanisms described in the previous section, often to a greater extent than their planktonic cell counterparts (Ricci, 2015). Another advantage of biofilms over planktonic cells is their higher resistance to antibiotics, leading to improved chance of survival in a competitive soil environment (Mah et al., 2003). This is an important consideration when applying microbial inoculants to soils where microbes face intense competition and may not be as well adapted to challenging conditions as indigenous soil microbes (Anderl et al., 2000; Mah and O’Toole, 2001; Whiteley et al., 2001; Donlan, 2002; Walters et al., 2003; Resch et al., 2005; Zhang and Mah, 2008; Beaudoin et al., 2012). An alternative mechanism by which biofilms enhance plant growth is through biocontrol of disease organisms (Innerebner et al., 2011), such as competitive colonization of the rhizosphere and the production of antimicrobial compounds (Bais et al., 2004; Lugtenberg and Kamilova, 2009; Chen et al., 2013).

The literature contains several examples of the PGPR activity of biofilms. Single and dual-species biofilms produced from *Pseudomonas, Trichoderma, Bradyrhizobium*, and *Penicillium* showed greater ammonia production, IAA production, phosphate solubilization, siderophore production, and/or nitrogenase activity than the planktonic inocula (Bais et al., 2004; Jayasinghearachchi and Seneviratne, 2004; Triveni et al., 2012; Mohd and Ahmad, 2014). Furthermore, when the biofilms were used to inoculate seeds, cotton seed germination, wheat root and shoot length, soybean dry weights and nitrogen accumulation, and maize seed germination and root length were increased compared to plants inoculated with planktonic cells (Mohd and Ahmad, 2014).

### Using Biochar to Promote Microbial Growth and Survival in Soil

Biochar has received much attention in the scientific literature over the last decade, as a soil amendment due to its ability to improve soil fertility and increase crop yields. Biochar can change soil fertility parameters that influence microbial survival in soil, including pH, organic matter content, cation exchange capacity and nutrient retention, water retention and oxygen tension, bulk density and provide niche spaces for microbes, thus preventing grazing by fungal predators (Major, 2009; Clough and Condron, 2010; Gaskin et al., 2010; Singh et al., 2010; Van Zwieten et al., 2010; Kameyama et al., 2012; Jaafar, 2014; Ye et al., 2016; Backer et al., 2017; Jenkins et al., 2017). Recent research has also investigated the use of biochar as a carrier material for microbial inoculants, applied as seed-coatings, constituting a sustainable alternative to peat-based inoculants, and promoting early colonization of the rhizosphere with beneficial microorganisms (Rondon et al., 2007; Budania and Yadav, 2014; Adam et al., 2016; Deb et al., 2016; Egamberdieva et al., 2016; Głodowska et al., 2016; Kim et al., 2016; Shanta et al., 2016; Siddiqui et al., 2016; Sun et al., 2016; Traxler et al., 2016; Nadeem et al., 2017; Vecstaudza et al., 2017). It is important to note, however, that not all biochar materials are the same; biochar production conditions and feedstock materials have a large influence on the biological, chemical and physical properties of the final biochar material and while many provide desirable effects on soil fertility, some can be toxic to microbes and/or plants (Nguyen et al., 2017; Wang et al., 2017).

### Challenges Moving From the Lab to the Field

While the technology of bio-inoculants holds a promising future, some major bottle necks have to be addressed to increase their efficacy. The use of PGPRs as inoculants is centuries old; the use of these inoculants have been largely focussed on legumes and cereals (Sessitsch and Mitter, 2015). Development of new PGPR inocula is based on laboratory screening assays that rely on specific PGPR mechanisms, namely nitrogen fixation, ACC deaminase activity, auxin synthesis and calcium phosphate solubilization. However, screening of pure culture isolates for those with PGPR functions does not always result in isolates that promote plant growth under field conditions. At the same time, those which have minimal *in vitro* growth promoting functions may have alternate mechanisms to promote plant growth. Since these mechanisms are less well-understood, they are difficult to screen for under laboratory conditions. As a result, beneficial strains that employ these mechanisms are discarded based on poor performance on classical *in vitro* PGPR screening methods (Cardinale et al., 2015).

Developing inocula containing highly effective microbes with a long shelf-life and high rhizosphere colonization rate poses a major challenge for commercialization. PGPR are often used to inoculate plant material without an appropriate carrier or in quantities that do not allow for efficient rhizosphere colonization under field conditions, due to competition with resident soil micro- and macro-fauna. In addition, soils growing high value crops are often fumigated with broad spectrum biocidal fumigants that alter the bio-community structure of the soil. Long-term fumigation affects soil microbes and their interactions that help plants with nutrient acquisition and mobilization, thereby affecting soil health (Dangi et al., 2017). This may also pose a challenge to rhizosphere colonization by PGPR inocula.

Plant breeding has been instrumental in the success of Green Revolution. However, in the context of bio-inoculants, very little has been done to integrate microbiome-based plant breeding to achieve a heritable PGPR community that enhances crop productivity (Mitter et al., 2013; Trivedi et al., 2017). The Green Revolution also has introduced inorganic fertilizers, pesticides, and herbicides into soils leading to extensive damage in the form of contaminants. Combining bioremediation with plant growth promotion would be a beneficial approach in addressing this global agriculture problem. Designing microbial consortia to address various aspects of bioremediation and plant growth potential is an essential aspect to this approach (Macouzet, 2016; Baez-Rogelio et al., 2017). Synthesis of bio-inoculants for specific soil conditions, to overcome environmental constraints, and training farmers and associated staff to efficiently apply them to crop plants is very important element in the development and deployment of more beneficial inocula (Bashan, 2016; Parnell et al., 2016; Itelima et al., 2018).

## Applications

Bacteria with multiple benefits can be advantageous in commercial agriculture and are relevant to the bio-economy. Many plants of economic significance are grown in monoculture and require amendments for optimal growth and yield, as well as protection against disease organisms (Vejan et al., 2016; Andreote and Pereira, 2017).

### Increasing Yield and Decreasing Fertilizer Inputs

Utilization of bacterial consortia has inconsistent effects on crop yield (Wu et al., 2009). The mixing of a bacterium (*B. amyloliquefaciens*) with a fungus (*Trichoderma virens*) improves yields of corn and tomato, among other crops (Akladious and Abbas, 2012; Molla et al., 2012) and is available in the market place. The company Excalibre-SA (ABM) combines *Trichoderma* with *Bradyrhizobium* for improved growth of soybean while BioGrow Endo (Mycorrhizal Applications) combines arbuscular mycorrhizal fungi and *Trichoderma* for improved growth and treatment of pathogens present in the soil; both of which are commercially available.

Inoculation with N-fixing bacteria (*Azospirillum* and *Azobacter*) allowed half-rate N-fertilizer application and increased sesame seed yield and oil quality (Shakeri et al., 2016). Similar effects were shown for *Azospirillum vinelandii* inoculated *Brassica carinata* cv. *Peela raya* (Nosheen et al., 2016a,b). A consortium of bacteria (*Bacillus cereus* PX35, *Bacillus subtilis* SM21, and *Serrati asp* XY2) reduced the incidence of root knot nematode (*Meloidogyne incognito*) in tomato, increased fruit yield (31.5 to 39%) and quality (soluble sugars, vitamin C, and titratable acids) (Niu et al., 2016).

Advanced biofuels are derived from non-food biomass (Ajjawi et al., 2017), often lignocellulosic material, to minimize any competition with food production; the long-term goal is provision of renewable fuels, along with high value bio-products, to reduce the atmospheric CO_2_ emissions associated with fossil fuels (Rokem and Greenblatt, 2015). Conversion of lignocellulosic material to fuel needs to become easier and less expensive to make this fuel economically competitive (Kuhad et al., 2011); in addition, there needs to be improved biomass availability from purpose-grown biomass crops (e.g., *Miscanthus*, switchgrass, and *Sorghum bicolour*) (Carpita and McCann, 2008; Lynd et al., 2008; Margaritopoulou et al., 2016; McCalmont et al., 2017). The growth and productivity of purpose grown biofuel crops can be improved through inoculation with PGPR (Smith et al., 2015a) as has been demonstrated for switchgrass (Ker et al., 2012, 2014; Shanta et al., 2016; Arunachalam et al., 2017). Marginal and contaminated lands can be used to grow biofuel crops in order to avoid conflicts around food versus energy crops. With the use of PGPR that contain natural potential to cope with soil contaminants, the biofuel crops could be used efficiently for phytoremediation and also to reduce high levels of agrochemicals residues in agriculture lands (Weyens et al., 2009b; Evangelou and Deram, 2014).

### Improving Disease Control and Reducing the Use of Agrochemicals

Biologicals are an alternative method for combating plant pathogens (Harman, 2000), and there are commercially available examples (Velivelli et al., 2014). Beneficial rhizobacteria may secrete antibiotics and other compounds antagonistic to plant pathogens. Production of antibiotics is one of the more common biocontrol mechanisms (Fravel, 1988; Doumbou et al., 2001; Compant et al., 2005). There are commercially available examples of biocontrol agents (Velivelli et al., 2014).

Pathogens often develop resistance to the antibiotics and other mechanisms of biocontrol, so that they cannot be fully controlled in the long-term. A holistic approach with multiple controlling methods is probably better than excessive dependency on a single solution when confronting pathogens. Over the long term, pathogen-antagonistic bacteria will also evolve their mode of action to counteract the pathogens. PGPR also produce antibiotics such as lipopeptides, polyketides and antifungal metabolites that suppress pathogens (Prashar et al., 2013).

## Roadmap to Commercialization

Bioformulations of the products for plant growth promotion, soil fertility and suppression of phytopathogens offer green alternatives to conventional agrochemicals (Arora et al., 2016). Agricultural products can be developed on the basis of live single- or multi-species inoculum or based on isolated signal molecules. In the case of signal compounds, one can use microbe-to-plant signals, for direct effects on the plants, or even plant-to-microbe signals to trigger enhanced production of the microbe-to-plant signals in the soil environment, assuming the presence of the microbe in the soil. One could also use plant-to-microbe signals to control the composition of the phytomicrobiome in ways that are beneficial to the crop plants.

The development of PGPR-based inoculants is not strictly defined but generally includes the following steps:

(1) Isolation of the bacteria from roots or other plant tissues.
(2) Laboratory and controlled growth environment screening.
(3) Field screening for a range of crops, geographic locations, planting dates and soil types.
(4) Evaluation of the possible combinations of strains and/or signals.
(5) Consideration of the management practices (e.g., agrochemical use and rotation)
(6) Refinement of the product.
(7) Experiments confirming absence eco-toxicological effects.
(8) Product delivery formulation – e.g., peat, granular, liquid or wettable powder.
(9) Registration and regulatory approval of the product.
(10) Product available on the market.

### Live PGPR Inoculum

For the development of a single-strain inoculum, one begins by isolating microbes from plants. This is achieved by extensive sampling of plants from a range of habitats (agricultural, dry, wet, cold, hot, and saline). Currently efforts are more focused on the rhizomicrobiome as it has the greatest microbial diversity. Once the cultivable strains have been isolated, they can be screened for ability to enhance germination of *Arabidopsis*, or crop plants. Promising isolates can then be screened for ability to accelerate emergence and early plant growth, under controlled environment conditions. Germination and early plant growth experimentation should be conducted under both optimal and stressful plant-growth conditions. In general, the easiest stress to apply uniformly is salt stress; salt stress responses are generally representative of responses expected for other stresses (Subramanian et al., 2016a,b). However, if a signal molecule responsible for effects on plant growth is a protein, saline conditions may denature it, rendering it ineffective; this is why experiments should also be conducted under optimal and other stressful conditions, time and resources permitting. The most promising PGPR can then be evaluated under the more complex and demanding conditions of the field, to select the top-performing strains for commercialization.

When screening for strains that control diseases (Weyens et al., 2009a; Wagner et al., 2014) Petri plate assays can be used to test for biocontrol activity against common plant pathogens. The disease strain is inoculated onto potato dextrose agar (PDA), and the PGPR strain is inoculated on a disk of filter paper to determine an inhibition or kill zone around the disk (Ilangumaran and Smith, 2017; Ilangumaran et al., 2017; Takishita et al., 2018). Results can be validated *in planta*, under controlled conditions and eventually under field conditions.

It is clear that some strains will be overlooked with this approach. Not all PGPR strains will be cultivable. In addition, there could be strains that do not show promising results at early stages (e.g., do not affect germination) but would enhance subsequent growth. However, given the large number of strains to evaluate at this stage, we must accept this risk and consider revisiting the situation once initial-stage screening is complete.

In the case of consortia, managing the strains so that they are in consistent proportions within the resulting product can be a challenge; combining the strains near or at the end of their growth cycles may result in the most reliable outcomes. However, consortia, through interactions among the strains, may well offer advantages over single strain-based inoculum.

### Signal Compound-Based Products

For strains showing promise, effective signal compounds, potentially biostimulants, can be isolated and developed into products. To do this, PGPR strains are grown in broth cultures and then the cells are removed through a combination of centrifugation and filtration (Gray and Smith, 2005; Gray et al., 2006). The supernatant can then be evaluated for the ability to promote seed germination and early plant growth, as described in the “Live PGPR inoculum” section. If the liquid promotes growth, then it can be concentrated and subjected to HPLC for fractionation. Fractions corresponding to peaks are collected and their ability to promote plant growth under controlled conditions, or biocontrol activity against a pathogen, using the Petri plate methods described in “Live PGPR inoculum” section. Once a given peak has demonstrated activity, the compound is isolated, purified and subjected to mass spectrometry and, possibly other chemical analyses, to determine its identity.

At this time, there are signal-based products on the market that use microbe-to-plant signals. In some cases, the signal molecule is produced on an industrial scale by cultivation of PGPR in the presence of a plant-to-microbe signal molecule which triggers the production of the microbe-to-plant signal molecule. For example, the production of LCOs by rhizobia can be triggered by addition of appropriate plant-to-microbe signals, generally isoflavonoids (Smith et al., 2015b), although in some cases jasmonates can also be used (Mabood et al., 2014). The addition of isoflavonoids to trigger LCO production has been developed as a technology and is now widely applied as a growth enhancement for a broad range of crops (Smith et al., 2017). Thuricin 17, a small protein produced by *B. thuringiensis* NEB17, and LCOs can both be extremely effective in mitigating the effects of abiotic stresses on a wide range of crop plants (Subramanian et al., 2016a,b). Thuricin 17 is in the early stages of being commercialized.

### Product Formulation, Registration and Intellectual Property

To generate PGPR- or signal compound-based products, formulations must be developed that allow for even distribution in the field. For example, the legume inoculant industry has focused on solid carriers, the most common of which is sterilized peat (Bashan et al., 2014), which is inoculated with cells, and adhered to seeds using sticking agent at the time of sowing. Due to concerns about sustainable sourcing of peat, alternative solid carriers such as alginate (Bashan, 2016) have been investigated. Recently, biochar has been shown to be a high potential alternative because its porosity and nutrient content can be altered according to source material and production conditions (Głodowska et al., 2016).

Alternatively, liquid inoculants can be sprayed onto seeds prior to sowing or dripped into the seed furrow at the time of sowing. Signal molecules are probably best applied as liquid sprays, although slow release solid formulations could also be investigated. The ones commercialized so far have been effective at very low concentrations, so the actual mass or volume of the signals *per se* is extremely low. Storage and product lifespan are important considerations that need to be determined for a given product, to ensure microbial survival and/or bioactivity of the strain or compound of interest.

Another consideration is acute versus chronic application of PGPR or signal molecules. Acute application occurs just once or a limited number of times during a growing season, on the seed or at a target stage of crop development, or in response to environmental conditions, such as onset of drought. In the case of chronic application, the product could be applied at regularly timed sprays or as a slow-release seed treatment.

As the product nears the marketplace, it is necessary to have approval for registration. In Canada, this often requires safety and efficacy data; the product must also meet other specific regulatory requirements. However, when the technology is very novel, it may not fit into pre-existing regulatory categories and therefore require the regulatory agency to conduct consultations. Important considerations include manufacturing practices and documentation of efficacy and safety from a third party.

Currently, the regulatory procedures for registration and commercialization of biostimulants are complex. The main reason for the absence of a specific harmonized framework for European Union, United States, and Canada, is that there is no standard legal or regulatory definition for plant biostimulants. Du Jardin (2015) proposed the following definition: “A plant biostimulant is any substance or microorganism applied to plants with the aim to enhance nutrition efficiency, abiotic stress tolerance and/or crop quality traits, regardless of its nutrients content.” This definition could be amended to include: By extension, plant biostimulants also designate commercial products containing mixtures of such substances and/or microorganisms.

The biostimulants currently available in Europe, are registered via two routes: (1) the European pesticides law which combines supranational and national provisions for introducing plant protection products on the national markets or (2) following the national regulations on fertilizers specific to each European state. In the United States, federal agencies (EPA and USDA) regulate registration of biostimulant products. Every state has its own set of compliance programs for their registration, which follow state-specific standards, fees and other mandates (Du Jardin, 2015). Presently every product submitted for registration in Canada, is considered as a unique product; therefore, every biostimulant is commercialized via its own pathway. When the product is sold it will be under a label with specific claims. If the claims are around enhanced nutrient uptake and other fertility aspects the product may be grouped with fertilizers and approval may be more straightforward. If the product is a biocontrol agent (Berendsen et al., 2012; Gu et al., 2016) with claims related to -cidal activity there will be additional scrutiny and time involved. It can be wise to claim fewer properties at the early stages of licensing to move more quickly to market, however, this may constrain the ability to claim further benefits after licensing. In terms of efficacy testing, if this is required, it may be good to have, at least in the later stages, on-farm testing, as this causes the grower community to be more engaged, which enhances acceptance and edges toward marketing.

Of course, underlying all stages of product development is the matter of intellectual property. One can no longer patent life forms or naturally occurring compounds, but formulations and uses can be patented (Matthews and Cuchiara, 2014). Thus, when a novel technology is possible, a patent search must be conducted. If there is freedom to operate (FTO) then a patent application can be filed; if enough supporting data is available, the full application can occur immediately. If time is required to produce supporting data, an application for a 1-year provisional patent can be submitted and followed by a full and formal patent application.

### Private-Public Partnerships for Increased Knowledge and Improved Training

Every step in the process from microbe isolation to licensing is laborious, expensive and requires time. Collaboration between industrial, academic and government research should become an important part of the product development process. Biotechnology organizations, for example, Genentech in South San Francisco, California encouraged their researchers to conduct side scientific projects and share their outcomes in publications. Universities are now pursuing commercialization of their innovation discoveries. Today, associations among companies and the scholarly world are common (Tachibana, 2013). As the sector develops there will be a need to train more experts in the area, through university research activities, often in collaboration with industry, as this brings the commercialization perspective to the research activities and imparts it to the trainee.

## Conclusion

The relationships between plants and the phytomicrobiome are ancient and represent the result of a very long coevolution. Evolution is pragmatic, random and relentless, and we should expect to discover many additional and sometimes surprising relationships that are beneficial to crops, and therefore global food production. It is clear that members of the phytomicrobiome offer huge potential in terms of new and more sustainable crop management practices, however, it is also clear that we understand only a tiny amount of this potential and a very great deal remains to be done.

Probably the easiest area for exploitation at the outset will be around single strains or consortia with small numbers of members and/or the signal compounds they produce. These could be focused on stimulation of plant growth, particularly under adverse conditions, such heat and drought stress, which are becoming increasingly prevalent as climate change progresses. Another set of products could be focused on plant disease control. We have examined the steps necessary to develop these technologies into products and have them approved for sale through the regulatory process.

Finally, one should take care to have “public license.” At this point the public perception of “bio” is not overly well formed, but generally positive. At the same time, there is public concern around the use of “chemicals” and biologicals are seen as a positive alternative, in the form of “plant probiotics.” It is our duty to try to anticipate any problems with phytomicrobiome technologies and to forestall their development, while projecting the benefits to the public. These technologies should be compliant with organic crop production practices and it would be useful to have them registered as such. The phytomicrobiome offers enormous potential for agricultural benefit, in terms of global food security, crop production sustainability and making agricultural systems climate change resilient. We need to ensure that this is approached in a systematic, thorough and broadly considered manner.

## Author Contributions

Each author contributed by generating drafts of specific sections of the manuscript and then participating in repeated editing of the manuscript as it moved toward its final form.

## Conflict of Interest Statement

The authors declare that the research was conducted in the absence of any commercial or financial relationships that could be construed as a potential conflict of interest.
